# The Magic Lantern as an Aid to Instruction in Chemical and Physical Lectures

**Published:** 1874-04

**Authors:** Hermann Vogel


### THE MAGIC LANTERN AS AN AID TO INSTRUCTION IN CHEMICAL AND PHYSICAL LECTURES.

BY PROF. HERMANN VOGEL.

Two years ago I called the attention to the use of the magic lantern in lectures, as has long been the custom in America in large and small educational institutions. The instrument permits small figures, whether pen sketches, wood cuts or photographs, from nature, two inches square, to be enlarged to four or five feet, so that in this way almost every wood cut in chemical and physical text books may be converted into a sort of wall chart, which can be conveniently exhibited to large audiences.

The construction of the instrument is very simple. It consists of three principal parts: First, the source of light, which in large lanterns consists of an electric or calcium light, in smaller ones of a petroleum lamp. Second, a condenser, as it is called, consisting of two plano-convex lenses of short curvature with the curved sides towards each other, D Q. Their only use is to concentrate the light upon the object magnified, for the stronger the illumination the more it will bear enlarging. Third, a system of magnifying lenses, usually a portrait objective, such as is employed by photographers.* This throws an enlarged picture of a small original, which is placed just in front of the condenser, on a screen formed of white paper or white muslin calsomined. The electric light is too troublesome for general use. The calcium light is more convenient, for the oxygen may be prepared before hand and preserved for weeks in rubber bags. Petroleum furnishes the most simple light, and, with a suitably arranged apparatus, is intense enough for a lecture room holding 50 to 70 persons, provided the pictures do not exceed 1 1-2 meters square. The petroleum lamps formerly used were unsatisfactory, but Talbot, 11 Karlstr., Berlin, has introduced a lantern with a petroleum light which surpasses all those previously constructed. The light was so intense that I was able to obtain an objective spectrum, eight inches long. For using this apparatus in the day time it is necessary to have tight shutters or thick curtains which can be closed.

The pictures for the magic lantern I have prepared either by photographing larger pictures or by fastening wood cuts printed on silk paper, or drawings made on such paper, directly to the glass by means of negative varnish (shellac dissolved in alcohol). The varnish is poured on a glass plate held in a horizontal position and the wood cut laid upon it, care being taken to avoid air bubbles, and held firm while the varnish flows off. When dry the plate is slightly warmed and again flowed over it, the plate being turned round as the varnish runs off. Suitable impressions of wood cuts can now be obtained only of the publishers of scientific works, but on the general introduction of the lantern they would soon come into market.

*The usual portrait lens consists of two double convex crown glass lenses, corrected by plano-concave flint lenses placed between them.

Not only can pictures be exhibited but certain interesting experiments shown, such as the rise of the thermometer in a solidifying solution of hyposulphite of soda, and the fall of temperature in a mixture of sulphocyanides and water. To prevent heating the solutions by the lamp, a cell containing water, or better an alum solution may be interposed.

The diagrams shown at the meeting of the Chemical Society on the 9th of November, by the aid of Talbot's lantern were partly photographs of Bunsen's diagrams, partly wood cuts from Roscoe's spectrum analysis, obtained from Fred. Vieweg and Son, in Braunschweig, partly the so-called relief prints on glass, made by Woodbury's photographic printing process, and partly original photographs from the spectrum itself.—*Journal of Applied Chemistry.*

